# Impact of Obesity on Long-Term Urinary Incontinence after Radical Prostatectomy: A Meta-Analysis

**DOI:** 10.1155/2018/8279523

**Published:** 2018-04-03

**Authors:** Yong Wei, Yu-Peng Wu, Min-Yi Lin, Shao-Hao Chen, Yun-Zhi Lin, Xiao-Dong Li, Qing-Shui Zheng, Xue-Yi Xue, Ning Xu

**Affiliations:** Department of Urology, First Affiliated Hospital of Fujian Medical University, Fuzhou 350005, China

## Abstract

Obesity is a known risk factor for prostate cancer progression and may contribute to poor treatment outcomes. However, little is known concerning the relationship between obesity (body mass index [BMI] *⩾* 30) and the urinary incontinence (UI) of patients after radical prostatectomy (RP). The goal of this study was to focus on the prevalence and duration of UI after RP with specific attention to the BMI. Subsequently, trials were identified in a literature search of PubMed, Embase, Cochrane Library, Web of Science, and Google Scholar using appropriate search terms. All comparative studies reporting BMI, study characteristics, and outcome data including the relationship between BMI and urinary incontinence data were included. Finally, four studies comprising 6 trials with 2890 participants were included. The results showed that obesity increased UI risk at 12 months in patients who underwent robotic-assisted laparoscopic radical prostatectomy (RLRP) (odds ratio [OR] 2.43, 95% confidence interval [CI] [1.21, 4.88], *P* = 0.01). When stratified by the surgical methods, the pooled results showed that obesity increased UI risk at 24 months in patients who underwent RLRP (OR 2.00, 95% CI [1.57, 2.56], *P* < 0.001). However, in patients who underwent laparoscopic radical prostatectomy (LRP), the pooled results showed that obesity does not increase UI risk at 24 months (OR 1.13, 95% CI [0.74, 1.72], *P* = 0.58). This is the first study to include obesity as the primary independent variable. Outcomes indicate that obesity (BMI ≥ 30) may increase the UI risk at 12 and 24 months after RLRP. Well-designed randomized controlled trials with strict control of confounders are needed to make results comparable.

## 1. Introduction

Worldwide, obesity has long been related to prostate cancer progression [1] and has become a growing health problem for the prevalence of global obesity which is increasing [2]. Therefore, urologists are going to meet more obese participants with prostate cancer in the near future. Recently, the advent of prostate-specific antigen (PSA) screening and increased public awareness accelerate the increasing number of radical prostatectomies (RP) [3]. However, urinary incontinence (UI) remains to be one of the most concerning complications. Therefore, some surgeons are trying to use body mass index (BMI) as a prognostic factor for determining which treatment to recommend [4]. To date, there is a lack of data in terms of the relationship between early continence at 1 month and long-term continence at 24 months and BMI after RP for prostate cancer patients [3, 5]. Our meta-analysis aimed to investigate the relationship between BMI and UI after RP for prostate cancer patients.

## 2. Materials and Methods

### 2.1. Ethical Approval

This article does not contain any studies with human participants performed by any of the authors.

### 2.2. Search Strategy

We searched PubMed, Embase, Cochrane Library, Web of Science, and Google scholar databases for articles published before November 12, 2016. A combination of search terms was used including “BMI”, “body mass index”, “prostate cancer”, “prostatectomy”, “incontinence”, and “continence”. The search was conducted with a language restricted to English publication.

### 2.3. Inclusion Criteria

The inclusion criteria were as follows: (1) original articles in English publication; (2) trials reporting individual demographic, UI, and BMI information and clinical follow-up data; (3) trials assessing the relationship between UI and BMI. Single arm trials, case reports, and systematic reviews were excluded.

### 2.4. Data Extraction and Quality Assessment

Two investigators (Yu-Peng Wu and Min-Yi Lin) extracted data, respectively, employing a predefined data extraction form. Subsequent full-text record screening was fulfilled independently by two investigators (Ning Xu and Yu-Peng Wu). Disagreements were resolved by a third reviewer (Yong Wei). All of included trials in our meta-analysis contained data as follows: first author's name, published year, number of patients, and preoperative parameters. We made several attempts to contact the corresponding authors to obtain the necessary data to meet inclusion criteria, when their studies did not meet inclusion requirements. At least 3 follow-up attempts were made for queries sent; unfortunately, these attempts were unsuccessful. The quality of each included study was evaluated by the Newcastle-Ottawa scale, which is widely used for assessing the observational studies.

### 2.5. Statistical Methods

Statistical analysis was conducted utilizing RevMan5.3. Chi-square and *I*-square tests were employed to test the heterogeneity of different trials; no heterogeneity existed when *P* > 0.1 and *I*^2^ < 50%; a fixed-effects model was applied to pool the trial results. Significant heterogeneity was identified if *P* < 0.1 and *I*^2^ > 50%, and a random-effects model was employed [6].

## 3. Results

### 3.1. Workflow of Literature Research

**Figure 1:**
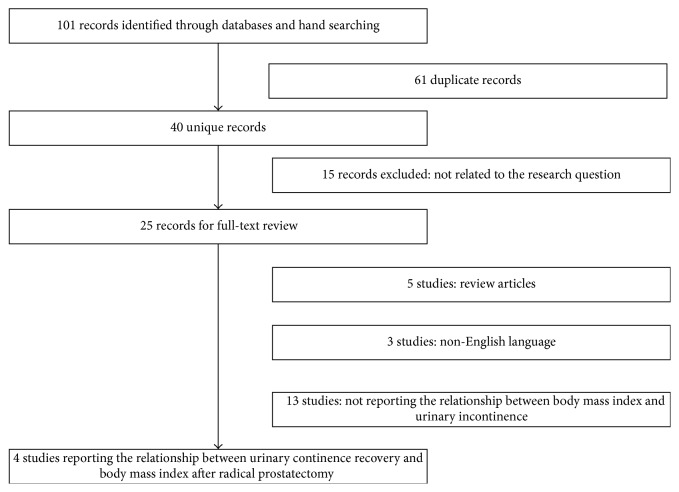
Flow diagram for selection of the included trials reviewed.

After primary literature search, 101 potential relevant studies were found and 61 duplicate studies were excluded. Then, after reading the title and abstract, 15 studies were further excluded. Finally, 21 additional studies were removed by two authors (Yu-Peng Wu and Min-Yi Lin) accessing the full text independently. Therefore, 4 studies [5, 7–9] were included in this meta-analysis. We described study procedures details in Figure 1. Two authors (Yu-Peng Wu and Min-Yi Lin) completed this work independently, and any disagreements were dealt with by discussion.

### 3.2. Study Characteristics

Four included studies recruited 2890 participants. The demographics of enrolled individuals and tumor characteristics are presented in Table 1.

### 3.3. Definition of UI

UI is bothersome complication after RP. However, when evaluating UI, the definition is one of the most important things. So far, there has been no clear definition of UI. The amount of pad use was selected for the definition of UI; however, how many pads as UI were different depends on the reports. If the definition is different, the conclusion is different. Thus, the different definitions of all included articles were listed below. Continence was defined as “completely dry” or the use of one safety pad and patients who used more than one protective pad daily were classified as incontinent by Gozen et al. [5]; Ahlering et al. [7], Wiltz et al. [9], and Brown et al. [8] defined urinary continence as “no pad.”

### 3.4. UI at 1 Month

**Figure 2:**
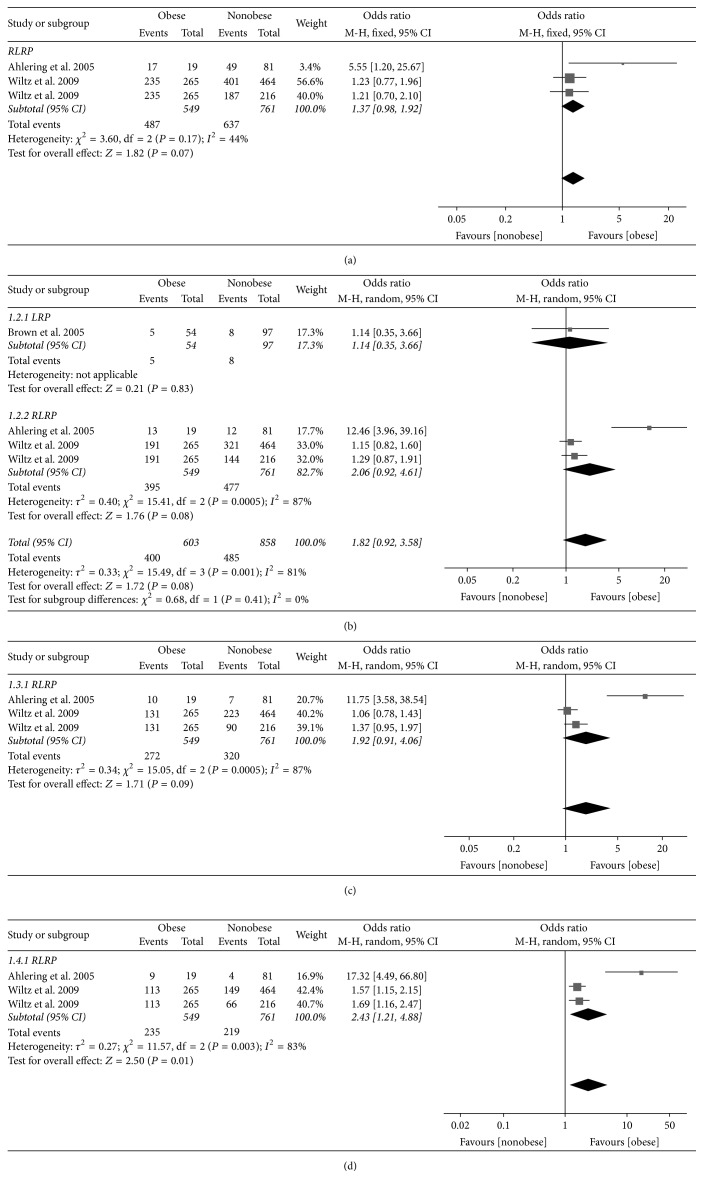
Forest plot comparing urinary incontinence rates between obese and nonobese men at 1 month (a), 3 months (b), 6 months (c), and 12 months (d).

Three trials reporting the UI data at 1 month consisted of 1310 participants. The overall pooled OR indicated that there was no significant association between obesity and UI in patients who underwent robotic-assisted laparoscopic radical prostatectomy (RLRP) (odds ratio [OR] 1.37, 95% confidence interval [CI] 0.98 to 1.92, *P* = 0.07) (Figure 2(a)).

### 3.5. UI at 3 Months

Four trials reporting the UI data at 3 months consisted of 1461 participants. The overall pooled OR indicated that there was no significant association between obesity and UI (OR 1.82, 95% CI 0.92 to 3.58, *P* = 0.08). Patients were then stratified by the surgical methods. In LRP subgroup, there was no significant association between obesity and UI (OR 1.14, 95% CI 0.35 to 3.66, *P* = 0.83). In RLRP subgroup, there was also no significant association between obesity and UI (OR 2.06, 95% CI 0.92 to 4.61, *P* = 0.08) (Figure 2(b)).

### 3.6. UI at 6 Months

Three trials reporting the UI data at 6 months consisted of 1310 participants. The overall pooled OR indicated that there was no significant association between obesity and UI in patients who underwent RLRP (OR 1.92, 95% CI 0.91 to 4.06, *P* = 0.09) (Figure 2(c)).

### 3.7. UI at 12 Months

Three trials reporting the UI data at 12 months consisted of 1310 participants. The overall pooled OR indicated that there was a significant association between obesity and UI in patients who underwent RLRP (OR 2.43, 95% CI 1.21 to 4.88, *P* = 0.01) (Figure 2(d)).

### 3.8. UI at 24 Months

**Figure 3:**
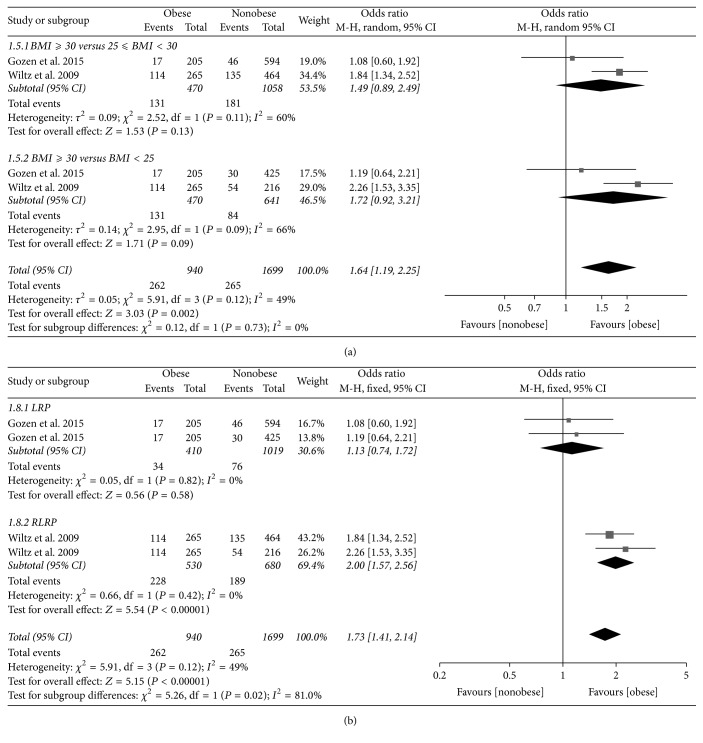
Forest plot comparing urinary incontinence rates between obese and nonobese men at 24 months stratified by body mass index (a) and surgical method including laparoscopic radical prostatectomy (LRP) and robotic-assisted laparoscopic radical prostatectomy (b).

Four trials reporting the UI data at 24 months consisted of 2639 participants. We performed a subgroup analysis on obesity; the pooled results indicated there was no significant association between obesity and UI in both BMI ≥ 30 versus 25 ≤ BMI < 30 (OR 1.49, 95% CI 0.89 to 2.49, *P* = 0.13) and BMI ≥ 30 versus BMI < 25 (OR 1.72, 95% CI 0.92 to 3.21, *P* = 0.09) subgroups. However, the overall pooled OR indicated obesity increased the risk of UI at 24 months in patients who underwent radical prostatectomy (OR 1.64, 95% CI 1.19 to 2.25, *P* = 0.002) (Figure 3(a)).

When stratified by the surgical methods including LRP and RLRP, in LRP subgroup, the pooled results showed that the obesity does not increase the risk of UI at 24 months (OR 1.13, 95% CI 0.74 to 1.72, *P* = 0.58). However, in RLRP subgroup, the pooled results indicated that the obesity increased the risk of UI at 24 months (OR 2.00, 95% CI 1.57 to 2.56, *P* < 0.001). The overall pooled results demonstrated that obesity increased the risk of UI at 24 months (OR 1.73, 95% CI 1.41 to 2.14, *P* < 0.001) (Figure 3(b)).

## 4. Discussion

Obese men have been found to be more likely to suffer from UI after RP. Populations of men with weight gain and central adiposity in adults are more likely related to a higher prevalence of lower urinary tract symptoms [10–12]. However, it is still controversial in terms of the relationship between obesity and UI after RP. Wolin et al. [13] showed that preprostatectomy obesity may be significant factor in UI after RP, and this result was consistent with previous studies by Kim et al. [14] and Kumar et al. [15]. Mao et al. [16] reported that BMI was independent predictor of postoperative UI at 3 months after surgery. Anast et al. [17] reported that BMI may contribute to the worse UI. A study of 100 patients by Ahlering et al. [7] reported that obese men demonstrated a longer urinary continence recovery time after RP. However, the study of Mulholland et al. [18] demonstrated that UI after RP was not related to patient BMI, and this result was also consistent with previous studies by Wallerstedt et al. [19]. Kadono et al. [20] also reported that there was no statistical difference in preoperative factors including BMI after RP. Therefore, this meta-analysis was performed to systematically evaluate the association between obesity and UI after RP. To the best of our knowledge, this study is the first meta-analysis with a focus on the relationship between obesity and UI.

Basiri et al. [21] performed a meta-analysis regarding UI between RLRP and LRP groups. The results revealed that the rate of UI was significantly lower after RLRP than LRP. Considering the efficacy of operative technology, subgroup analysis stratified by LRP and RLRP was performed. After combining results from 4 studies consisting of 6 trials, our meta-analysis demonstrated that there was a significant relationship between obesity and UI at 12 and 24 months in patients who underwent RLRP. However, there was no significant association between obesity and UI at 24 months in patients who underwent LRP.

Currently, there is a lack of data in terms of predictors of 1-month UI after RP for prostate cancer patients. Our results showed that early UI 1 month following RP was not related to BMI. The reason may contribute to the fact that both obese patients and nonobese patients underwent stressful and tiring times in the early postoperative period. Lavigueur-Blouin et al. [3] reported that BMI was not an independent predictor of continence at 1 month on multivariate analysis, which is in accord with our meta-analysis results. Obese men often possess a larger prostate volume, which means that a larger prostate volume was associated with urinary continence. Konety et al. revealed that prostate volume was a predictor of recovery of urinary continence after RP. Lower level of continence up to 2 years after RP was observed in men with larger prostate volume.

Mao et al. [16] showed that BMI was an independent predictor of UI at 3 months after prostatectomy. However, our meta-analysis revealed that there was no significant difference between BMI and UI at 3 months. Mizutani et al. [22] showed that no significant association was observed 3 months after RP. Ahlering et al. [7] revealed a significant difference in continence rates between obese and nonobese group at 6 months. However, our meta-analysis revealed that there was no significant difference between BMI and UI at 6 months. The mechanisms responsible for the discrepancy are unclear; further research is required.

Although there are lots of studies that focus on UI after RP, this meta-analysis is the first to include obesity as the primary independent variable. However, there are several limitations of our study. First of all, the prevalence of postprostatectomy UI can be influenced by many kinds of factors, including the participant preoperative parameters, the experience of surgeons, different kinds of techniques used by surgeons, and data collected and reported using different methods [23–25]. With the development of society, RP techniques have changed and improved over time. The publication year of 4 studies included in this meta-analysis varied from 2005 to 2015; it is difficult to assess the potential difference in techniques in statistical models. Secondly, data about the length of follow-up after treatment for determination of biochemical failure was missing. Thus, we did not mention it in this study. Third, in terms of the small sample size and limited number of studies enrolled, the results may lack statistical power. Further studies need to be done in the near future. Thirdly, the current meta-analysis only considers BMI. Thus, other studies of adiposity including waist circumference were not included in the current meta-analysis.

## 5. Conclusions

In conclusion, this study indicated that obesity may increase the risk of UI at 12 and 24 months in patients who underwent RLRP. However, there was no significant association between obesity and UI at 24 months in patients with LRP. The results should be confirmed by well-designed randomized controlled trials with strict control of confounders to make results comparable.

## Figures and Tables

**Table 1:** Demographic and clinical data of obese and nonobese patients.

	Study									
	Gozen et al.			Brown et al.		Wiltz et al.			Ahlering et al.	
	Normal: BMI < 25	Overweight: BMI 25–30	Obese: BMI > 30	Nonobese: BMI < 30	Obese: BMI > 30	Normal: BMI < 25	Overweight: BMI 25–30	Obese: BMI > 30	Obese: BMI > 30	Nonobese: BMI < 30
Patients	425	594	205	97	54	216	464	265	19	81
Age (years)	64.1 (40–82)	63.9 (44–84)	63.5 (49–75)	58 ± 6	57 ± 6	60.3 ± 7.1	59.7 ± 6.5	59.4 ± 6.2	62.6 (53–70)	62.3 (43–78)
Preoperative PSA (ng/mL)										
Mean	-	-	-	6.2 ± 4.1	6.8 ± 3.8	6.3 ± 5.1	6.4 ± 3.9	6.4 ± 4.3	7.4 (0.1–21.9)	8.1 (1.1–62)
<4 ng/ml	50 (12)	71 (12)	27 (13)	-	-	42 (19)	92 (20)	58 (22)	-	-
4–10 ng/ml	223 (52)	318 (53)	108 (53)	-	-	155 (72)	313 (67)	178 (67)	-	-
>10 ng/ml	152 (36)	205 (34)	70 (34)	-	-	19 (9)	59 (13)	29 (11)	-	-
Preoperative Gleason score										
<7	191 (56)	260 (56)	93 (59)	79 (85)	41 (79)	141 (65)	294 (63)	168 (63)	44 (54)	11 (58)
7	122 (36)	172 (37)	55 (35)	12 (13)	5 (10)	58 (27)	138 (30)	83 (31)	26 (32)	5 (26)
8–10	29 (8)	32 (7)	9 (6)	2 (2)	6 (12)	17 (7)	32 (7)	14 (6)	11 (14)	3 (16)
Clinical stage										
cT1	110 (26)	119 (20)	31 (15)	71 (76)	43 (84)					
cT2	211 (50)	296 (50)	95 (46)	22 (24)	8 (16)					
cT3	104 (24)	179 (30)	79 (39)	-	-					
cT1c						181 (84)	342 (74)	186 (70)	-	-
cT2a						25 (12)	89 (19)	62 (23)	-	-
cT2b/c						10 (4)	33 (7)	17 (7)	-	-
Surgical technology	LRP			LRP		RLRP			RLRP	
Operation time (min)	197 (102–465)	210 (113–450)	219 (110–484)	192 ± 34	208 ± 43	217 ± 58	214 ± 65	234 ± 77	295.8 (186–645)	236.1 (160–490)
EBL (mL)	727 (100–1010)	767 (100–1080)	904 (300–1090)	-	-	199 ± 152	215 ± 203	231 ± 172	183 (50–400)	105 (25–350)
Nerve sparing										
No	242 (57)	382 (64)	153 (75)	-	-	9 (4)	32 (7)	26 (10)	5 (26)	13 (16)
Unilateral	61 (14)	86 (15)	25 (12)	-	-	52 (24)	120 (26)	74 (28)	5 (26)	20 (25)
Bilateral	122 (29)	126 (21)	27 (13)	-	-	155 (72)	312 (67)	165 (62)	9 (48)	48 (59)
PSM	79 (18.6)	127 (21.4)	60 (29.3)	22 (23)	12 (23)	37 (17)	79 (17)	59 (22)	3 (16)	22 (27)
Catheterization (days)	7 (5–28)	7 (4–27)	7 (4–17)	-	-	6.0 ± 1.1	6.0 ± 1.3	6.0 ± 1.8	-	-
Hospital stay (days)	10 (5–23)	11 (4–20)	11 (4–25)	2.1 ± 1	2.1 ± 1.2	1.2 ± 0.6	1.2 ± 1.2	1.2 ± 1.5	41 (18–96)	28.4 (18–168)
Complications	55 (12.9)	84 (14.1)	27 (13.2)	16 (16)	4 (7)	22 (10.4)	49 (10.9)	37 (14.5)	5 (26.3)	4 (4.9)
Postoperative Gleason score										
<6	238 (57)	305 (53)	96 (47)	71 (74)	36 (69)	130 (60)	246 (53)	140 (53)	12 (63)	43 (53)
7	143 (34)	229 (40)	86 (43)	25 (26)+	16 (31)+	71 (33)	189 (41)	109 (41)	6 (32)	24 (30)
8–10	39 (9)	40 (7)	20 (10)	-	-	15 (7)	29 (6)	16 (6)	1 (5)	14 (17)
Pathological stage										
T0/2	273 (64)	338 (57)	112 (55)	81 (84)	42 (79)	180 (83)	373 (80)	204 (77)	16 (84)	59 (73)
pT3/4	152 (36)	236 (43)	93 (45)	15 (16)	11 (21)	36 (17)	91 (20)	61 (23)	3 (16)	22 (27)
Biochemical recurrence	74 (17.4)	101 (17)	33 (16.1)	3 (5)	2 (6)	12 (1.5)	20 (2.5)	13 (1.6)	-	-

BMI = body mass index, PSM = positive surgical margin, EBL = estimated blood loss, LRP = laparoscopic radical prostatectomy, and RLRP = robotic-assisted laparoscopic radical prostatectomy.
